# The role of exercise-mediated mitochondrial quality control remodeling in aging

**DOI:** 10.3389/fcell.2026.1792645

**Published:** 2026-03-31

**Authors:** Tao Cai, Yating Li, Yunsong Zhang, Chunyan Li, Shanhui Li, Qi Zhang

**Affiliations:** 1 School of Sports Medicine and Health, Chengdu Sport University, Chengdu, China; 2 Sports Medicine Key Laboratory of Sichuan Province, Chengdu, China; 3 Affiliated Sports Hospital of Chengdu Sport University, Chengdu, China; 4 Institute of Sports Medicine and Health, Chengdu Sport University, Chengdu, China; 5 Hejiang County Hospital of Traditional Chinese Medicine, Luzhou, China; 6 Hejiang Hospital of Traditional Chinese Medicine Affiliated to Southwest Medical University, Luzhou, China

**Keywords:** age, aging, exercise, mitochondrial quality control, physical training

## Abstract

Aging is intimately associated with multisystem functional decline and an increased risk of chronic diseases. A pivotal cytological basis underlying this process is the progressive dysregulation of the mitochondrial quality control (MQC) network. Emerging evidence suggests that MQC is not a singular process but rather a multitiered synergistic system encompassing mitochondrial biogenesis, dynamic remodeling, selective autophagy (mitophagy), proteostasis maintenance, and coordinated mitochondrial–organelle communication. This integrated network is critical for preserving cellular energy homeostasis, redox balance, and stress tolerance. During aging, impairments in mitochondrial genomic coordination, network topology, autophagic flux, and protein import and folding collectively contribute to bioenergetic decline, chronic low-grade inflammation, and metabolic imbalance. As a safe and sustainable nonpharmacological intervention, regular exercise systematically remodels MQC structure and function by integrating signaling axes such as AMPK, SIRT1, and p38 MAPK, thereby promoting coordinated mitochondrial renewal and partially reversing aging-associated mitochondrial dysfunction. On the basis of a systematic elucidation of the core mechanisms of MQC and its dysregulation during aging, this review highlights the differential regulatory effects of distinct exercise modalities—specifically endurance training, high-intensity interval training (HIIT), and resistance training—on mitochondrial dynamics, autophagic flux, proteostasis, and mitochondrial turnover. Furthermore, the intrinsic associations among exercise–MQC coupling, inflammatory responses, metabolic imbalances, and emerging peripheral biomarkers are explored. Finally, current research limitations and challenges in clinical translation are analyzed, and future research directions regarding dose–response relationships, multimodal exercise prescriptions, personalized strategies, and systemic integrated regulation are proposed. This review aims to provide a refined theoretical basis for optimizing exercise-based anti-aging interventions.

## 1 Introduction

Aging is a systemic biological process characterized by the progressive disruption of tissue homeostasis, and its progression is closely associated with an increased risk of chronic conditions such as neurodegenerative diseases, metabolic syndrome, and cardiovascular diseases (López-Otín et al., 2023). With the deepening of research in mitochondrial biology, accumulating evidence indicates that the role of mitochondria in aging extends far beyond the traditional view that regarded them merely as “passive victims” of reactive oxygen species–induced damage. Current perspectives propose that mitochondria actively drive the initiation and progression of aging-related functional decline through a complex regulatory network encompassing biogenesis, dynamics, selective autophagy, and proteostasis, collectively referred to as mitochondrial quality control (MQC). Dysregulation of this network results in a progressive decline in mitochondrial function, leading to disturbances in energy metabolism, activation of inflammation, and reduced cellular stress tolerance, ultimately manifesting as tissue dysfunction and increased disease susceptibility (Szklarczyk et al., 2014; Banarase et al., 2023).

In contrast to earlier research paradigms that emphasized the accumulation of mitochondrial damage, recent studies increasingly focus on understanding the dynamic coordination among the various mechanisms of MQC at a systems level. Mitochondrial biogenesis, dynamics, and mitophagy are regarded as an integrated functional network, and their coordinated imbalance may precede multisystem functional decline (Koklesova et al., 2022; Gottlieb et al., 2021). Under healthy conditions, MQC maintains the stability of mitochondrial population structure and function through complementary mechanisms of renewal, selection, and clearance (Holt, 2010). However, with advancing age, declines in the quality of newly generated mitochondria, network fragmentation, impaired autophagic flux, and the accumulation of misfolded proteins become exacerbated, rendering cells more susceptible to energy deficiency and chronic low-grade inflammation, thereby accelerating the aging process (Feng and Lu, 2025; Skawratananond et al., 2025).

Regular physical exercise represents a well-established and readily implementable non-pharmacological intervention for delaying aging (Grolaux et al., 2024; Lohman et al., 2023). Although early explanations primarily focused on exercise-induced increases in mitochondrial content and biogenesis, emerging evidence suggests that the protective effects of exercise largely depend on its multilayered regulation of the mitochondrial quality control system (Craige et al., 2024; Díaz‐Castro et al., 2024). Exercise enhances mitochondrial renewal efficiency and adaptive capacity through the integration of multiple signaling pathways, thereby partially reversing aging-related mitochondrial dysfunction.

Despite continuous advances in research, several key questions remain unresolved, including how different MQC components achieve effective coordination under exercise-induced stress; whether specific exercise modalities exert preferential regulatory effects on particular MQC pathways; and how mechanistic insights at this level can be translated into clinically generalizable exercise prescription strategies. In this context, the present review systematically integrates current evidence regarding the role of exercise-mediated MQC remodeling in aging. We first delineate the structural and functional logic of MQC, identify critical nodes of dysregulation during aging, and summarize the regulatory characteristics of different exercise modalities. On this basis, we further analyze the association between tissue-level mechanisms and systemic adaptations, and explore how this association relates to aging-associated inflammation and metabolic imbalance. It should be clarified that the majority of existing literature focuses on skeletal muscle at the mechanistic level, as it is metabolically active, highly responsive to exercise stimuli, and serves as a classical tissue model for investigating exercise-induced MQC remodeling (Choi et al., 2025). However, exercise-triggered MQC adaptations are not confined to local muscle but are accompanied by systemic signal reprogramming. In recent years, peripheral samples—including blood, plasma/serum, peripheral blood mononuclear cells (PBMCs), platelets, extracellular vesicles, and circulating cell-free mitochondrial DNA (ccf-mtDNA)—have increasingly been utilized to assess exercise-related mitochondrial adaptations, and the corresponding indicators to some extent reflect alterations in whole-body mitochondrial homeostasis and inflammatory status. In addition to skeletal muscle tissue, this review also incorporates systemic peripheral biomarkers into the discussion. Finally, we address current limitations in the field and challenges in clinical translation, and propose directions for future research. We anticipate that this review will facilitate an integrated understanding of the relationships among exercise, mitochondrial quality control, and aging, and provide a reference framework for optimizing anti-aging intervention strategies.

## 2 Methodology

### 2.1 Literature search strategy

A systematic search was conducted in the PubMed and Elsevier ScienceDirect databases. The search period was defined from 2015 to 2025. The search strategy combined controlled vocabulary terms and free-text keywords, which were integrated using Boolean logical operators. Reference lists of relevant review articles and key research studies were manually screened, and citation tracking was performed to supplement potentially overlooked important publications, thereby ensuring comprehensive literature coverage. The search string was as follows: (“exercise” OR “physical training” OR “endurance training” OR “high-intensity interval training” OR “resistance training”) AND (“mitochondrial quality control” OR “mitochondrial biogenesis” OR “mitochondrial dynamics” OR “mitophagy” OR “autophagy” OR “mitochondrial unfolded protein response”) AND (“aging” OR “ageing” OR “sarcopenia” OR “frailty”)

### 2.2 Inclusion and exclusion criteria

The inclusion criteria were as follows:

1. Explicit inclusion of exercise interventions or structured physical training protocols;
2. Assessment of at least one MQC-related indicator, including but not limited to: markers of mitochondrial biogenesis; proteins associated with mitochondrial dynamics; indices related to mitophagy; respiratory chain complex activity or mitochondrial respiratory function parameters;
3. A research context involving aging.

The exclusion criteria were as follows:

1) Studies limited to *in vitro* cellular experiments without an organismal aging context; 2) Studies that did not report quantifiable MQC-related outcome measures; 3) Non-English publications; 4) Conference abstracts lacking complete supporting data.

### 2.3 Literature screening process

Two investigators independently conducted title and abstract screening. Full texts of preliminarily eligible studies were subsequently retrieved for further evaluation. Discrepancies arising during the screening process were resolved through discussion, and when necessary, adjudicated by the corresponding author.

### 2.4 Quality assessment

**FIGURE 1:**
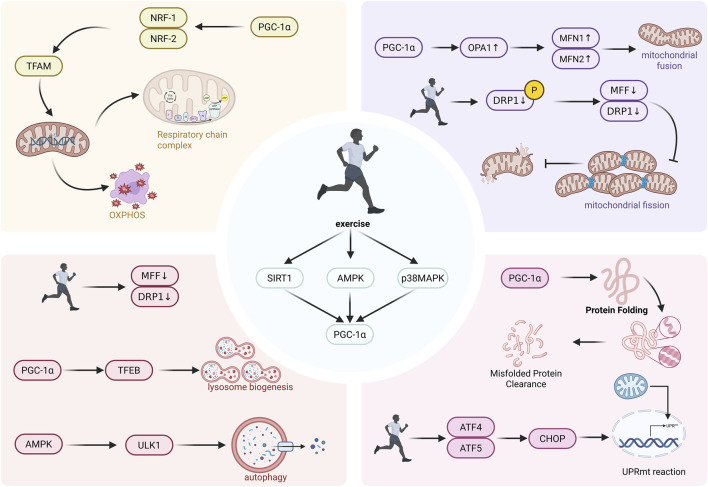
Structural and functional basis of mitochondrial quality control (MQC).

To evaluate the quality of the included studies, we applied a modified version of the Grading of Recommendations Assessment, Development and Evaluation (GRADE) approach. This method was used to assess risk of bias, methodological rigor, and the strength of evidence for each study. High-quality studies (e.g., well-controlled randomized trials and studies with large sample sizes) were prioritized in the evidence synthesis (Figure 1).

## 3 Structural and functional foundations for maintaining mitochondrial quality control homeostasis

### 3.1 Cross-genomic coordination safeguards the intrinsic quality of mitochondrial biogenesis

Mitochondrial biogenesis constitutes the initiating step of mitochondrial quality control (MQC). It integrates multiple signals, including cellular energy status, redox homeostasis, and nutrient sensing, thereby determining the structural integrity, metabolic capacity, and long-term adaptive potential of newly formed mitochondria (Scarpulla, 2012; Kaur and Naqvi, 2025). Peroxisome proliferator-activated receptor gamma coactivator-1 alpha (PGC-1α) serves as the central transcriptional coactivator within this regulatory network (Dumesic et al., 2025; Gidlund et al., 1985; Moissidis et al., 2025). Upon sensing cellular energy stress and redox fluctuations, PGC-1α is activated through post-translational modifications, such as AMP-activated protein kinase (AMPK)-mediated phosphorylation and sirtuin 1 (SIRT1)-mediated deacetylation. Activated PGC-1α subsequently translocates to the nucleus, where it cooperates with nuclear respiratory factors to initiate the transcription of genes encoding respiratory chain subunits, fatty acid oxidation enzymes, and factors involved in mitochondrial DNA (mtDNA) replication and transcription, thereby systemically activating the mitochondrial biogenesis program (Clark and Parikh, 2021; Ke et al., 2021; Uittenbogaard and Chiaramello, 2020).

Concurrently, PGC-1α drives the coordinated expression of nuclear-encoded respiratory chain subunits and mitochondrial transcription factor A (TFAM) by activating transcription factors such as NRF1/2 and ERRα. TFAM represents a critical node in coordinating transcription between the nuclear and mitochondrial genomes. By binding to the mtDNA control region and inducing conformational changes, TFAM plays an essential role in transcription initiation, replication, and structural packaging. The co-expression of oxidative phosphorylation (OXPHOS) subunits encoded by both nuclear and mitochondrial genomes maintains the proper stoichiometry of respiratory chain complexes. The assembly of these complexes depends on the coordinated integration of subunits encoded by the dual genomes; dysregulation of either genome can disrupt this homeostatic balance, leading to defective respiratory chain assembly, abnormal mitochondrial architecture, and functional impairment (Hees and Harbauer, 2022; Cuppari et al., 2019; Papier et al., 2022).

During aging, although certain tissues may exhibit transient compensatory upregulation of PGC-1α signaling, the persistent accumulation of mtDNA mutations, progressive decline in mitochondrial translational machinery, and structural impairment of supercomplex assembly mechanisms collectively disrupt mitochondrial proteostasis and compromise the functional integrity of the OXPHOS system. These defects may reduce the capacity of newly generated or remodeled mitochondria to establish membrane potential, diminish oxidative phosphorylation efficiency, and weaken antioxidant stress resistance, thereby exacerbating aging-associated declines in energy metabolism (De Smalen et al., 2023; Safdar et al., 2016; Parmar et al., 2024).

### 3.2 Spatial organization and network topology shape the platform for mitochondrial quality control

Beyond molecular composition, the subcellular spatial distribution and network topology of mitochondria profoundly influence their functional performance and MQC efficiency (Wai and Langer, 2016; Pickles et al., 2018; Steffan et al., 2025). Such structural heterogeneity establishes the basis for spatial recognition and functional compartmentalization in subsequent selective clearance mechanisms. Distinct mitochondrial subpopulations exhibit significant differences in anatomical localization and functional responsibilities. For example, in skeletal muscle, mitochondria demonstrate clear subcellular functional specialization: subsarcolemmal mitochondria (SSMs) are primarily distributed near capillaries and perinuclear regions, facilitating nutrient and oxygen sensing and predominantly participating in substrate uptake and metabolic regulation; in contrast, intermyofibrillar mitochondria (IFMs), characterized by a high surface area-to-volume ratio and tight embedding within the sarcomeric structure, are responsible for rapid ATP generation and localized energy supply to sustain continuous contractile function (Willingham et al., 2021).

In most eukaryotic cells, mitochondria form dynamically interconnected networks through fusion and fission. This connectivity facilitates the uniform distribution of electrochemical gradients, metabolites, and signaling molecules across the mitochondrial population, thereby enhancing systemic functional robustness. However, in mature skeletal muscle cells, mitochondria are spatially constrained by myofibrils and the cytoskeleton, aligning along sarcomeres to form a distribution pattern tightly coupled to contractile units. Their degree of network connectivity varies according to muscle fiber type and is particularly heterogeneous in oxidative fibers (Holt et al., 2024; Katti et al., 2022; Milner et al., 2000).

Aging systemically disrupts tissue structural order, resulting in the loss of subcellular localization specificity and fragmentation of the mitochondrial network. These alterations are associated with decreased activity of fusion proteins, increased activity of fission proteins, weakened cytoskeletal support, alterations in mitochondria-associated membrane structures, and lipid peroxidation (Smith et al., 2023; Ma et al., 2024; Zorov et al., 2019). Disintegration of the network structure not only impairs the efficiency of energy and repair signal transmission across the mitochondrial population but also renders mitochondria more susceptible to diverse stressors.

### 3.3 Cooperative quality control mechanisms of mitochondrial dynamics and autophagy

Mitochondrial dynamics, through the dynamic balance between fusion and fission, enable real-time surveillance and quality selection within the mitochondrial network. Mitochondrial fusion is mediated by outer membrane proteins MFN1 and MFN2 and the inner membrane protein OPA1, promoting the exchange of mitochondrial contents and facilitating functional complementation, damage repair, and maintenance of metabolic homeostasis. In contrast, mitochondrial fission is executed by dynamin-related protein 1 (DRP1), which is recruited to the mitochondrial surface by membrane receptors such as MFF and MiD49/51. A critical function of fission is to actively segregate severely dysfunctional mitochondria from the network, generating independent depolarized fragments, thereby preventing damage propagation and creating conditions for subsequent clearance (Kleele et al., 2021; Alavi and Fuhrmann, 2013; Song et al., 2024). Aging frequently leads to an imbalance in mitochondrial dynamics, characterized by reduced expression or activity of OPA1 and MFN1/2, accompanied by enhanced DRP1 signaling, resulting in network fragmentation and loss of continuity. These structural alterations are often associated with impaired respiratory function, decreased membrane potential stability, and increased production of reactive oxygen species (ROS), particularly in highly metabolic tissues such as the heart and skeletal muscle (Sukhorukov et al., 2024; Tokuyama et al., 2022; Leija et al., 2022).

Regulation of mitochondrial dynamics lies between biogenesis and autophagic degradation, determining whether damaged mitochondria undergo repair or are targeted for removal. Dysfunctional mitochondria identified through dynamic selection are primarily processed via PINK1–Parkin pathway–mediated selective autophagy (mitophagy). Upon loss of mitochondrial membrane potential, PINK1 kinase stabilizes on the outer mitochondrial membrane, subsequently recruiting and activating the E3 ubiquitin ligase Parkin, which induces extensive ubiquitination of outer membrane proteins. These ubiquitin chains serve as “eat-me” signals recognized by autophagy adaptor proteins, ultimately targeting damaged mitochondria for encapsulation and delivery to lysosomes for degradation (Xu et al., 2025; Jiménez-Loygorri et al., 2024). However, aging also impairs this terminal clearance pathway, resulting in a generalized decline in autophagic flux. Even when mitochondrial damage is successfully marked by the PINK1–Parkin system, the efficiency of subsequent steps—including autophagosome formation, trafficking, and fusion with lysosomes—is markedly reduced (Ordureau et al., 2020; Shan et al., 2025; Chen et al., 2023).

**FIGURE 2:**
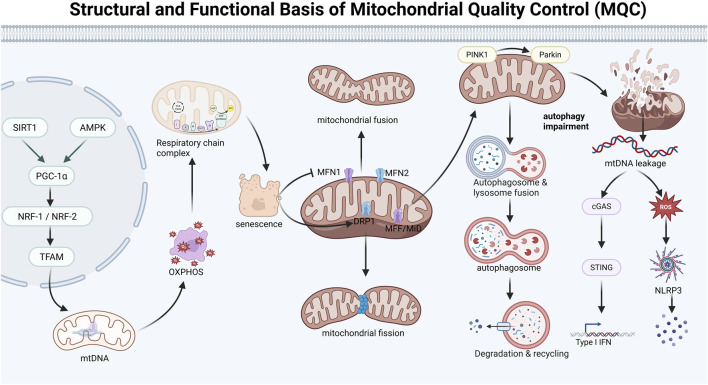
Synergistic mechanisms of exercise in regulating the dysregulation of mitochondrial quality control during aging.

Reduced autophagic clearance capacity leads to the accumulation of dysfunctional mitochondria and their contents within the cytoplasm. Notably, mitochondrial DNA (mtDNA) leaked into the cytosol can be recognized by the pattern recognition receptor cyclic GMP-AMP synthase (cGAS), thereby activating the cGAS–STING signaling pathway and inducing innate immune responses, including type I interferon production. Meanwhile, mtDNA and excessive ROS may function as danger-associated molecular patterns that promote the assembly and activation of the NLRP3 inflammasome, driving the maturation and secretion of pro-inflammatory cytokines such as interleukin-1β (IL-1β) (Vringer and Tait, 2023; Xian et al., 2022; Xu et al., 2022). Animal model studies further confirm that under conditions of Parkin deficiency–induced mitophagy impairment, inflammasome activation is more pronounced and closely associated with aggravated tissue injury (Li et al., 2019; Wang et al., 2024). These findings indicate that diminished autophagic efficiency is not only a driving factor for the accumulation of mitochondrial dysfunction but also a critical link connecting organelle dysfunction to systemic chronic inflammatory states (Figure 2).

## 4 Coordinated mechanisms by which exercise modulates mitochondrial quality control dysregulation during aging

### 4.1 Reactivation of upstream signaling networks attenuated in aging

With advancing age, the PGC-1α–centered mitochondrial regulatory network in skeletal muscle exhibits functional decline, manifested by reduced levels of key proteins such as PGC-1α and TFAM, as well as diminished activity of the upstream energy sensor AMPK. Exercise—particularly intermittent exercise accompanied by pronounced metabolic fluctuations—induces periodic perturbations in intracellular metabolite levels and calcium signaling, thereby activating signaling pathways including AMPK, p38 MAPK, and CaMKII. Within this regulatory network, AMPK acts as a primary energy sensor and is activated early; it cooperates with p38 MAPK to activate PGC-1α *via* phosphorylation and simultaneously elevates intracellular NAD^+^ levels to stimulate the deacetylase activity of SIRT1. SIRT1 further enhances the transcriptional coactivator function of PGC-1α through deacetylation (Chen WK. et al., 2018; Kang et al., 2013; Combes et al., 2015). This coordinated modification restores the mitochondrial gene expression program in aged muscle, promoting mitochondrial protein synthesis and functional improvement.

Activated PGC-1α broadly coordinates downstream gene expression, not only facilitating coordinated transcription between nuclear and mitochondrial genomes to drive mitochondrial biogenesis, but also upregulating genes related to fatty acid oxidation and antioxidant defense. Following long-term exercise training, the mitochondrial network in skeletal muscle undergoes structural and functional remodeling, characterized by increased mitochondrial density, enhanced respiratory chain complex activity, and improved overall oxidative phosphorylation capacity. In this process, reactive oxygen species (ROS) exert dual regulatory roles: on the one hand, exercise-induced ROS function as key signaling molecules that positively regulate exercise adaptation through activation of pathways such as MAPK; on the other hand, the antioxidant defense system enhanced in parallel with PGC-1α activation mitigates oxidative damage while preserving the signaling function of ROS, thereby re-establishing cellular homeostasis at an elevated metabolic level (Iijima et al., 2025; Halling et al., 2017).

### 4.2 Remodeling mitochondrial dynamics and optimizing damage segregation

In aged skeletal muscle and other tissues, the overall efficiency of the mitochondrial quality control system declines. Studies have shown that under stress conditions, the mitochondrial fusion protein OPA1 is prone to proteolytic cleavage and inactivation, thereby inhibiting the fusion of damaged mitochondria with the healthy network; meanwhile, DRP1-mediated fission signaling becomes relatively dominant, promoting the segregation of dysfunctional mitochondrial fragments from the network. This imbalance between fusion and fission is considered a major mechanism leading to mitochondrial network fragmentation (Marzetti et al., 2025a).

Regular exercise, through activation of the AMPK/SIRT1–PGC-1α axis, increases the expression of fusion proteins such as OPA1 and MFN1/2, while suppressing phosphorylation of Drp1 at Ser616 and reducing the recruitment of its adaptor proteins FIS1/Mff. These effects restrain excessive fission, enabling fragmented mitochondria to be reutilized and re-integrated into a highly interconnected network structure, thereby facilitating the sharing of membrane potential and matrix contents across the mitochondrial population, restoring oxidative phosphorylation efficiency, and contributing to the delay of aging phenotypes such as sarcopenia (Campos et al., 2023; Moore et al., 2019; Ruegsegger et al., 1985; Fealy et al., 1985; Long et al., 2022).

Restructuring of mitochondrial dynamics may provide the necessary structural foundation for subsequent selective clearance. Evidence indicates that exercise-induced selective mitophagy is frequently accompanied by mitochondrial fission. Exercise primarily activates the AMPK–ULK1 signaling axis and may also involve the PINK1/Parkin pathway, thereby promoting the labeling of damaged or dysfunctional mitochondria for entry into the autophagic process. Concurrently, exercise enhances the nuclear translocation and transcriptional activity of transcription factor EB (TFEB), promotes lysosomal biogenesis, and increases cellular degradative capacity. These processes help ameliorate the impaired autophagic flux commonly observed in aged tissues, reduce intracellular retention of damaged mitochondria, and consequently lower the risk of mtDNA leakage and mtDNA-mediated inflammatory responses. Under exercise intervention, mitochondrial dynamic balance and autophagic clearance mechanisms exhibit coordinated interactions, collectively maintaining mitochondrial population quality and functional homeostasis (Laker et al., 2017; Zhou et al., 2025; Huang et al., 2019).

### 4.3 Restoration of mitochondrial protein import and folding homeostasis

Aging is accompanied not only by abnormalities in mitochondrial morphology but also by marked disruption of mitochondrial proteostasis. Studies have demonstrated widespread downregulation of the mitochondrial translation program in aged muscle, resulting in reduced abundance of respiratory chain subunits (De Smalen and Handschin, 2025). As a potent stimulus, exercise promotes mitochondrial biogenesis while simultaneously regulating the synthesis, transport, and assembly of related proteins through pathways such as the PGC-1α/ERRα axis, thereby supporting renewal of the mitochondrial proteome. At the molecular level, exercise activates the highly conserved mitochondrial unfolded protein response (UPRmt), which cooperates with the mitochondrial quality control system to maintain organelle homeostasis. Experimental evidence indicates that both acute and chronic exercise induce the expression of key transcription factors, including ATF5, CHOP, C/EBP-β, and ATF4. These factors may translocate to the nucleus and coordinately upregulate downstream molecular chaperones (e.g., mtHsp70 and Hsp60) and proteases (e.g., ClpP and LONP1), thereby promoting proper protein folding and facilitating degradation of damaged proteins. In addition, the JNK pathway plays an important regulatory role in exercise-triggered UPRmt signaling (De Smalen et al., 2023; Gaspar et al., 2023). Furthermore, mitochondrial protein quality control is disrupted to varying degrees in aging, skeletal muscle disuse, and Parkinson’s disease (PD) models. In PD models, mitochondrial protein translocation mechanisms are markedly impaired, characterized by reduced expression of outer membrane translocases (TOM20/TOM40) and the inner membrane channel TIM23. This impairment is associated with abnormal accumulation of α-synuclein, which may bind to TOM20 and inhibit the import of nuclear-encoded proteins. In aged skeletal muscle, the import rate of matrix proteins may not necessarily decline; however, the stability of cytosolic precursor proteins is reduced, suggesting impairment at pre-import stages, and aging attenuates exercise-induced adaptive enhancement of protein import. Exercise intervention may ameliorate these processes through multiple mechanisms. In PD models, long-term treadmill exercise reduces α-synuclein accumulation and restores TOM/TIM expression. In other neuroprotective models, exercise-induced upregulation of Caveolin-1 contributes to maintenance of TOM20 levels and preservation of protein import function. Collectively, these changes promote efficient transport of nuclear-encoded proteins into mitochondrial subcompartments, alleviate cytosolic proteotoxicity, and restore mitochondrial homeostasis (Koo et al., 2017; Pan et al., 2021; Zhang et al., 2020).

In summary, exercise synergistically enhances multiple processes—including protein import, folding maintenance, and terminal clearance—thereby reconstructing and preserving mitochondrial proteostasis at the molecular level (see Table 1 for details). This mechanism complements exercise-mediated regulation of mitochondrial biogenesis, dynamics, and autophagy pathways, collectively forming a multilayered and highly adaptive quality maintenance network. These findings provide a systematic mechanistic framework for understanding how exercise delays aging-associated mitochondrial dysfunction.

**TABLE 1:** Exercise-mediated modulation of mitochondrial quality control (MQC) in aging.

Section	Core MQC module	Aging-associated dysregulation	Exercise-induced molecular changes	Functional outcomes	Study subjects	Level of evidence	References
3.1 Reactivation of Blunted Upstream Signaling Networks in Aging	PGC-1α–mediated mitochondrial biogenesis axis	Decreased PGC-1α mRNA and nuclear protein levels; reduced p-CREB; decreased TFAM, cytochrome c, and mtDNA/nDNA ratio	Increased nuclear PGC-1α protein; enhanced p-AMPK and p-p38 activity; restoration of TFAM expression, mtDNA content, and cytochrome c levels	Restoration of mitochondrial biogenesis and protein synthesis; attenuation of functional decline; reduced BAX/Bcl-2 ratio and Caspase-3 expression	Rats	Level III	Kang et al. (2013)
	AMPK–SIRT1 signaling synergy and anti-inflammatory network	Myocardial hypertrophy and elevated inflammation; downregulation of SIRT1/PGC-1α; chronic low-grade inflammation and reduced metabolic flexibility	Upregulation of SIRT1, PGC-1α, and AMPKα1; increased FOXO3a phosphorylation; improved insulin signaling; suppression of inflammatory responses	Improved myocardial architecture; enhanced antioxidant defense; restored insulin sensitivity and metabolic homeostasis	Rats; Humans (MetaMEx database analysis)	Level II–III	Chen et al. (2018a)
	Lipid metabolic reprogramming and intramyocellular lipid regulation	Myofiber degeneration and intramyocellular lipid accumulation	Modulation of lipid metabolism targets (PPARG, ADIPOQ, FABP4); enhanced fatty acid oxidation; inhibition of lipogenesis	Suppression of muscular fat infiltration; improved metabolic microenvironment	Humans (MetaMEx database analysis)	Level II	Iijima et al. (2025)
3.2 Remodeling of Mitochondrial Dynamics and Optimization of Damage Segregation	PGC-1α–dependent inhibition of mitochondrial fission and network remodeling	Excessive mitochondrial fragmentation; increased FIS1/DRP1 expression; Drp1 activation associated with metabolic impairment	Downregulation of FIS1 and DRP1; reduced Drp1 Ser616 phosphorylation; increased OPA1 expression; exercise effects partially dependent on PGC-1α	Restoration of mitochondrial reticular network; improved oxidative metabolism; amelioration of insulin resistance	Mice; Humans	Level II–III	Halling et al. (2017), Fealy et al. (1985)
	AMPK-mediated remodeling of mitochondrial dynamics and maintenance of adaptive capacity	Imbalance in mitochondrial fission–fusion cycling; aging-associated decline in exercise capacity	AMPK activation of dynamic cycling; tissue-specific regulation of Drp1 expression	Delayed age-related mitochondrial fragmentation; preserved exercise capacity and metabolic homeostasis	*Caenorhabditis elegan*s; Mice; Humans	Level II–III	Campos et al. (2023), Moore et al. (2019)
	Activation of the mitophagy–lysosome axis	Accumulation of dysfunctional mitochondria; reduced autophagic flux; impaired lysosomal function	AMPK-dependent phosphorylation of Ulk1 (Ser555); enhanced targeting of damaged mitochondria to lysosomes; AMPK–SIRT1–mediated TFEB nuclear translocation; mechanotransduction and myokine-mediated autophagy regulation	Clearance of damaged mitochondria; enhanced autophagic flux and lysosomal activity; maintenance of systemic homeostasis	Mice; Humans	Level II–III	Laker et al. (2017), Zhou et al. (2025), Huang et al. (2019)
3.3 Restoration of Mitochondrial Protein Import and Proteostatic Homeostasis	PGC-1α/ERRα-mediated recovery of mitochondrial translation	Reduced mitochondrial translational capacity; decreased PGC-1α and ERRα expression	Induction of the PGC-1α/ERRα axis; upregulation of mitochondrial ribosomal and translational proteins	Correction of mitochondrial protein synthesis defects; amelioration of sarcopenia-associated functional decline	Mice	Level III	de Smalen et al. (2023)
	JNK-driven mitochondrial unfolded protein response (UPRmt)	Downregulation of UPRmt-related gene expression	JNK activation; upregulation of Hspd1, LONP1, Yme1L1, ClpP, and ATF5	Enhanced skeletal muscle oxidative capacity; improved aerobic exercise performance	Mice	Level III	Gaspar et al. (2023)
	Restoration of mitochondrial protein import machinery (TOM/TIM complex)	Decreased TOM-40, TOM-20, and TIM-23 expression; α-synuclein accumulation	Increased TOM-40, TOM-20, and TIM-23 expression; upregulation of COX-I/IV; reduced α-synuclein accumulation	Attenuated dopaminergic neuronal loss; improved motor coordination in Parkinsonian models	Mice	Level III	Koo et al. (2017)

Level I —— Randomized controlled trials in human populations.

Level II —— non-randomized human studies or longitudinal intervention studies.

Level III ——mechanistic studies limited to animal or cell experiments.

### 4.4 The value of peripheral biomarkers in exercise and MQC research

Traditional assessment of mitochondrial quality control (MQC) has primarily relied on tissue biomarkers obtained through invasive procedures such as skeletal muscle biopsy, including measurements of mitochondrial respiratory enzyme activity or protein expression levels. However, the invasive nature of these approaches limits their applicability for repeated sampling in clinical populations and large-scale longitudinal studies. In recent years, peripherally derived biomarkers have attracted increasing attention as complementary tools for evaluating systemic mitochondrial homeostasis and stress status, thereby providing a novel perspective for exploring the relationship between exercise adaptation and mitochondrial function.

Specifically, tissue biomarkers mainly reflect organelle homeostasis within local muscle cells, whereas circulating biomarkers are more indicative of systemic mitochondrial stress levels and inter-organ communication. For example, circulating cell-free mitochondrial DNA (ccf-mtDNA) exists in multiple forms in the bloodstream, including naked mtDNA fragments, complexes bound to TFAM, fragments encapsulated within extracellular vesicles (EVs) or mitochondria-derived vesicles (MDVs), components of neutrophil extracellular traps (NETs), and a small proportion of intact mitochondria (Zhao et al., 2025; Nidadavolu et al., 2023). This structural heterogeneity suggests that ccf-mtDNA may originate from distinct biological processes, arising either from passive release during apoptosis or necrosis, or from regulated extrusion of mitochondrial contents, the latter being considered a component of MQC (Caicedo et al., 2024; Zhang et al., 2021; Fu et al., 2025; Trumpff et al., 2021).

Notably, the previously described mechanisms whereby mtDNA leakage activates the cGAS–STING and NLRP3 inflammatory pathways may be systemically reflected by alterations in circulating ccf-mtDNA levels. In other words, ccf-mtDNA may partially represent the process by which intracellular mitochondrial homeostatic imbalance is translated into systemic inflammatory signaling. In studies of aging and cardiometabolic diseases, elevated ccf-mtDNA levels have been associated with systemic inflammation, frailty status, and reduced exercise tolerance, with some evidence suggesting predictive value independent of traditional inflammatory markers (Mengozzi et al., 2025; Byappanahalli et al., 2023). The effects of exercise on ccf-mtDNA appear to be context-dependent. Acute high-intensity exercise may induce a transient increase in circulating levels, possibly related to acute mitochondrial stress and tissue remodeling responses during exercise. In contrast, long-term regular training is often accompanied by reduced resting ccf-mtDNA levels, suggesting improvement in chronic inflammatory status and enhancement of mitochondrial homeostasis. Therefore, when investigating the impact of exercise on MQC adaptation, differences in exercise modality and physiological status must be considered.

Beyond circulating DNA, peripheral blood mononuclear cells (PBMCs) and platelets provide additional information for assessing mitochondrial function. These cells are relatively easy to obtain and suitable for repeated sampling in research settings. Studies have shown that PBMC mitochondrial respiratory parameters—such as basal respiration, maximal respiration, and spare respiratory capacity—are associated with cognitive function, hippocampal volume, and white matter integrity (Qin et al., 2024; Dang et al., 2025; Mahapatra et al., 2023). Although these indices cannot fully substitute for direct assessment of skeletal muscle tissue, they offer reference information regarding systemic redox status and dynamic changes in energy metabolism.

Exercise-induced muscle adaptations may also be communicated systemically *via* extracellular vesicles (EVs). Myokines released during muscle contraction—including peptides, microRNAs (miRNAs), mtDNA, and proteins—can be transported through EVs such as exosomes, enter the circulation, reach distal organs (e.g., the brain), and be internalized by target cells through endocytosis (Memme et al., 2021). Meanwhile, MDVs and circulating EVs containing mitochondrial components may serve as peripheral manifestations of early MQC events, participating in inter-tissue signaling by carrying respiratory chain subunits or mtDNA (König and McBride, 2024; Chen et al., 2024). In addition, fibroblast growth factor 21 (FGF21) and growth differentiation factor 15 (GDF15), which are associated with mitochondrial stress responses, are upregulated in various tissue-specific models of mitochondrial dysfunction. Their secretion is linked to the mitochondrial unfolded protein response and the integrated stress response. Upon entering the circulation, these factors exert endocrine regulatory effects and participate in the modulation of energy metabolism. Physiological stimuli such as exercise training and cold exposure can likewise increase their circulating levels. Existing evidence indicates that plasma concentrations of FGF21 and GDF15 are associated with metabolic status and the degree of mitochondrial stress, suggesting their potential utility as auxiliary indicators for evaluating mitochondrial functional state and related metabolic adaptations (Jena et al., 2023).

**FIGURE 3:**
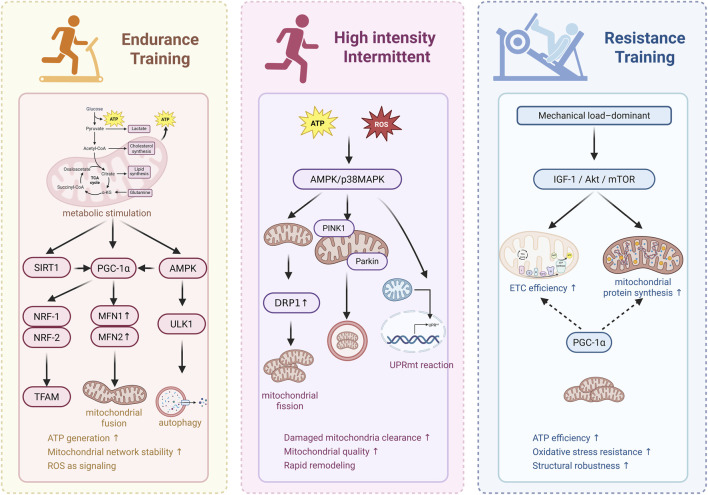
Mechanisms by which Different Exercise Modes Regulate MQC to Delay Aging.

In addition to regulating mitophagy and mitochondrial dynamics, exercise induces the secretion of specific myokines that participate in local and systemic signaling. Studies have demonstrated that the peptide hormone apelin, produced in response to muscle contraction, declines with aging, and its circulating concentration is positively correlated with muscle function in older adults. Systemic or muscle-specific deletion of apelin or its receptor APLNR exacerbates age-related muscle atrophy and strength decline, whereas exogenous apelin supplementation partially improves muscle fiber cross-sectional area and muscle function in aged mice by activating the AMPK pathway, promoting mitochondrial biogenesis and autophagic flux, and suppressing inflammatory responses. Moreover, apelin may act on muscle stem cells (MuSCs), enhancing their proliferative and differentiative capacities, thereby facilitating regenerative repair in aged muscle (Vinel et al., 2018) (Figure 3).

## 5 Mechanisms by which different exercise modalities regulate mitochondrial quality control to delay aging

### 5.1 Endurance training promotes mitochondrial renewal and reconstruction of homeostasis

Endurance training is typically characterized by moderate intensity and prolonged duration per session. Through periodic cumulative loading, it exerts mild yet sustained metabolic stimulation on the organism. Evidence indicates that this persistent aerobic metabolic stress effectively promotes mitochondrial biogenesis in skeletal muscle, optimizes mitochondrial network architecture, and enhances the long-term adaptive capacity of mitochondrial quality control (MQC) pathways. For example, in previously sedentary older adults, a 4-month endurance training program increased skeletal muscle mitochondrial content, predominantly accompanied by enhanced mitochondrial fusion. In contrast, lifelong endurance-trained athletes exhibited a molecular adaptation pattern characterized by mitophagy dominance and suppression of fission. These findings suggest that endurance training–induced remodeling of mitochondrial quality control is time-dependent (Arribat et al., 2019).

At the molecular level, long-term endurance training activates the AMPK–SIRT1–PGC-1α signaling axis and cooperatively upregulates PGC-1α expression and activity *via* pathways such as p38γ MAPK. As a central regulator of mitochondrial biogenesis, activation of PGC-1α not only promotes mitochondrial DNA replication and protein synthesis but also enhances fatty acid oxidation capacity and angiogenesis in muscle tissue, thereby improving mitochondrial respiratory function and antioxidant defense. These adaptive changes may partially delay or reverse aging-associated declines in PGC-1α expression and mitochondrial dysfunction. Furthermore, reactive oxygen species generated during endurance training can act as signaling molecules that participate in the regulation of mitochondrial-related gene expression through epigenetic mechanisms, including DNA methylation, histone modification, and miRNA expression (Kang et al., 2013; Halling et al., 2019; Gavin et al., 2015; Damal Villivalam et al., 2021; Caporossi and Dimauro, 2024). Concurrently, endurance training increases levels of nicotinamide phosphoribosyltransferase (NAMPT), restores the cellular NAD^+^ pool, and activates the mitochondrial deacetylase SIRT3, thereby enhancing antioxidant capacity and stabilizing respiratory chain function to provide metabolic support for long-term mitochondrial homeostasis (De Guia et al., 2019; Gibril et al., 2024).

At the ultrastructural level, endurance training synchronously optimizes mitochondrial network morphology and its contact interface with the endoplasmic reticulum—namely, mitochondria-associated membranes (MAMs)—thereby strengthening inter-organelle functional coupling. Typical features include upregulation of fusion proteins Mfn1/2 and OPA1 and suppression of excessively active fission protein Drp1, which reduces mitochondrial network fragmentation, improves cristae structural integrity, and lowers the risk of reactive oxygen species leakage and oxidative phosphorylation uncoupling (Zhang et al., 2025). In aged muscle, shortening of MAM structures is often accompanied by reduced calcium and metabolite exchange between organelles, potentially contributing to muscle functional decline. Endurance training can partially prevent such structural degeneration, maintaining MAM length and coverage, thereby optimizing calcium signaling and metabolic communication between the endoplasmic reticulum and mitochondria. It should be noted that although proteins such as Mfn2 may exhibit compensatory upregulation in aging, adaptive changes in a single molecule are insufficient to reverse the overall trend of MAM dysfunction and impaired energy coupling. By coordinately regulating multiple targets, endurance training stabilizes mitochondrial network connectivity, reduces fragmentation, and improves inter-organelle dynamic coupling, thereby enhancing mitochondrial calcium handling capacity, reducing reactive oxygen species leakage, and improving energetic efficiency (Wyckelsma et al., 2017; Alizadeh Pahlavani et al., 2022; Li et al., 2024; Allen et al., 2025).

At the level of autophagic regulation, regular endurance exercise upregulates autophagy-related proteins (e.g., Parkin) and maintains mitochondrial population quality through selective clearance of damaged mitochondria. Parkin-mediated selective autophagy represents an important effector mechanism for exercise-induced mitochondrial renewal. Long-term training reduces cellular dependence on autophagic clearance by continuously improving mitochondrial quality; however, this optimization process itself depends on intact Parkin function to ensure effective accumulation and preservation of functional mitochondria (Romanello and Sandri, 2015; Balan et al., 2019; Chen CCW. et al., 2018). In addition, endurance training activates transcription factor EB (TFEB), promotes lysosomal biogenesis, and enhances mitophagic flux, thereby facilitating timely removal of dysfunctional mitochondria (Marzetti et al., 2025a).

Overall, endurance training represents a homeostasis-oriented intervention that, through mild yet sustained metabolic and signaling stimulation, reduces the risk of energy fluctuation and mitochondrial functional mismatch. It is particularly suitable for populations requiring long-term management of aging-related metabolic imbalance and decline in mitochondrial quality. Beyond the mechanisms described above, endurance training may exert synergistic effects when combined with other interventions. For instance, n-3 polyunsaturated fatty acids (EPA/DHA) can be incorporated into the phospholipid bilayer of the mitochondrial inner membrane, altering its physical properties and protein function. Evidence suggests that such incorporation reduces reactive oxygen species production in aged skeletal muscle mitochondria and improves age-related impairment in ADP sensitivity. Based on current data, it has been proposed that endurance training and n-3 polyunsaturated fatty acid supplementation may act on mitochondria through complementary mechanisms—inducing mitochondrial network remodeling and optimizing membrane function, respectively—thereby producing additive or synergistic effects on improving energy metabolic efficiency (Broome et al., 2024).

Recent studies further indicate that denervation-induced muscle atrophy is accompanied by decreased mitochondrial respiratory function and increased reactive oxygen species production, alongside activation of the NF-κB pathway and its downstream NLRP3 inflammasome, caspase-1, and Gasdermin D–mediated pyroptotic signaling, as well as upregulation of STING protein expression. Aged muscle exhibits a similar pro-inflammatory phenotype. Notably, 6 weeks of endurance training significantly suppressed protein expression of NLRP3 and GSDMD in aged mouse muscle, showed a trend toward reduced caspase-1 and STING expression, decreased cytosolic mtDNA levels, and improved muscle mass, oxygen consumption, and exercise tolerance. These findings suggest that endurance training may exert anti-inflammatory effects through modulation of mitochondria–immune interactions (Khemraj et al., 1985).

### 5.2 High-intensity interval training enhances clearance and rapid remodeling

Compared with traditional endurance training, high-intensity interval training (HIIT) is characterized by alternating short bouts of maximal effort and recovery periods, resulting in pronounced yet transient fluctuations in energy metabolism and redox status. Such fluctuations rapidly increase the AMP/ATP ratio and generate acute peaks in reactive oxygen species, thereby activating key stress-responsive signaling pathways, including AMPK and p38 MAPK, and transcriptionally upregulating mitochondrial biogenesis–related factors such as PGC-1α (Zhang et al., 2025). Notably, even in sedentary older adults, a single acute HIIT session can induce phosphorylation of p38 MAPK and short-term upregulation of PGC-1α mRNA, suggesting that aged muscle retains a fundamental signaling responsiveness to high-intensity stimuli (Cobley et al., 2012). Recent evidence further indicates that sprint interval exercise markedly activates the mitochondrial unfolded protein response (UPR^mt^) and the integrated stress response, implying a distinctive role of HIIT in triggering protein quality control mechanisms (Botella et al., 2025).

At the level of mitochondrial dynamics and selective autophagy (mitophagy), HIIT induces phased dynamic remodeling. Acute high-intensity stimuli are often accompanied by increased phosphorylation of DRP1 at Ser616, promoting mitochondrial fission and segregating dysfunctional or depolarized fragments from the network (Zhang et al., 2025). Within these isolated fragments, PINK1 is more readily stabilized and recruits the E3 ubiquitin ligase Parkin, initiating PINK1/Parkin-mediated selective mitophagy to facilitate recognition and removal of damaged mitochondria (Moreira et al., 2017). Compared with moderate-intensity continuous training (MICT), some studies in aged skeletal muscle models have shown that HIIT can increase levels of PINK1 and Parkin, elevate the LC3-II/LC3-I ratio, and reduce accumulation of the autophagy adaptor protein p62 (Han et al., 2022). These molecular alterations suggest that HIIT may transiently enhance activation of autophagy-related pathways. Repeated long-term high-intensity training may also partially maintain or restore mitochondrial content in aged muscle through periodic activation of biogenic signaling; however, whether these changes translate into stable long-term reconstruction of mitochondrial function remains to be validated by longitudinal investigations (Coletti et al., 2022).

Parallel to mitochondrial dynamics is the regulation of proteostasis. HIIT-induced short-term fluctuations in the “mitochondrial–nuclear gene product ratio” can trigger UPR^mt^, upregulating chaperones and proteases such as LONP1 and HSP60 to assist in protein folding and repair (Co et al., 2021; Picca et al., 2023a). Animal studies further suggest that, compared with MICT, HIIT induces more pronounced changes in autophagy-related protein expression in certain models (Li et al., 2018). In addition, HIIT appears to fine-tune selectivity within autophagic pathways. For instance, HIIT significantly attenuates excessive activation of chaperone-assisted selective autophagy (CASA) induced by exhaustive exercise. Following exhaustive exercise, skeletal muscle shows upregulation of HSP70, BAG3, p62, and LC3-II, indicating recruitment of the CASA pathway for clearance of damaged proteins. However, in individuals preconditioned with HIIT, expression levels of these proteins, as well as the number of autophagosomes and autolysosomes, are markedly reduced. This phenomenon has been interpreted as reflecting a more balanced allocation of cellular resources between protein clearance and structural maintenance after training adaptation, rather than a mere amplification of degradative intensity (Lu et al., 2023).

Importantly, the effects of HIIT extend beyond peripheral skeletal muscle and involve adaptive regulation within the central nervous system. HIIT-induced elevations in peripheral and cerebral lactate allow lactate to cross the blood–brain barrier *via* monocarboxylate transporters, functioning directly as a signaling molecule to modulate mitochondrial quality control in the hippocampus. Studies demonstrate that lactate-mediated HIIT increases expression of mitochondrial fusion proteins OPA1, MFN1, and MFN2 in the hippocampus, while suppressing fission proteins DRP1 and FIS1, thereby optimizing the balance of mitochondrial dynamics. These structural improvements further promote upregulation of mitochondrial biogenesis signaling and brain-derived neurotrophic factor (BDNF) expression in the hippocampus, suggesting potential value of HIIT in delaying brain aging and associated cognitive decline (Hu et al., 2021).

### 5.3 Resistance training enhances structural integrity and functional stability

Resistance training primarily activates anabolic signaling pathways such as the IGF-1/Akt/mTOR axis through mechanical loading. Its direct physiological objective is to increase muscle fiber cross-sectional area and enhance muscle strength and functional reserve (Liu et al., 2021). Although traditionally considered to exert limited direct effects on mitochondrial quality control (MQC), emerging evidence indicates that resistance training induces a series of adaptive changes that support mitochondrial function, particularly by enhancing cellular structural stability, maintaining proteostasis, and improving the functional efficiency of existing mitochondria.

At the molecular and cellular levels, resistance training has been shown to increase mitochondrial complex IV activity, enhance electron transport chain efficiency, and reduce electron leakage and consequent oxidative stress. It also elevates the activity of antioxidant enzymes such as catalase and superoxide dismutase, thereby protecting mitochondrial structure from oxidative damage. Moreover, resistance training increases skeletal muscle mitochondrial sensitivity to ADP, improves respiratory function and energy conversion efficiency, and may positively influence the dynamic balance of mitochondrial networks. Collectively, these adaptations help mitigate age-related mitochondrial functional decline, counteract sarcopenia, and maintain overall metabolic health (Parry et al., 2020; Zhao and Wu, 2023; Mesquita et al., 2021; Flensted-Jensen et al., 2026).

From a regulatory perspective, resistance training may exhibit a distinct adaptive bias compared with endurance training. Animal studies indicate that resistance training preferentially induces a specific splice variant of PGC-1α, namely, PGC-1α4, which promotes protein synthesis and suppresses myostatin *via* the IGF-1/Akt pathway, thereby primarily facilitating muscle hypertrophy. Its effects on mitochondria are more related to functional optimization rather than simple volumetric expansion (Romanello and Sandri, 2015). Unlike the canonical PGC-1α1 isoform, PGC-1α4 does not significantly regulate genes involved in mitochondrial oxidative phosphorylation nor directly promote mitochondrial biogenesis. This mechanism explains why resistance training can induce myofiber hypertrophy without necessarily triggering comprehensive upregulation of mitochondrial gene expression programs, instead favoring optimization of existing mitochondrial functional efficiency to support increased mechanical load demands (Ruas et al., 2012).

Notably, the impact of resistance training extends beyond peripheral muscle tissue and shows potential benefits for the nervous system. Studies in mouse models of Alzheimer’s disease indicate that resistance training attenuates neuroinflammation and related pathology while improving mitochondrial health in the hippocampus. Long-term intervention studies further confirm that resistance training increases expression of electron transport chain proteins and dynamics-related regulatory molecules in aged skeletal muscle (Mesquita et al., 2020). In recent years, understanding of resistance training mechanisms from epigenetic and multi-pathway synergy perspectives has deepened. One study reported that 6 weeks of resistance training induced widespread demethylation of mitochondrial DNA (mtDNA) in skeletal muscle of older men, particularly within the D-loop region critical for mtDNA replication and transcription, where methylation levels were significantly reduced. These epigenetic changes were associated with increased mitochondrial gene expression and elevated protein levels of respiratory chain complexes III and IV, suggesting that resistance training may partially counteract age-related epigenetic alterations at the transcriptional level and participate in regulation of mitochondrial quality (Ruple et al., 2021).

Similarly, a report demonstrated that 10 weeks of resistance training significantly upregulated mRNA expression of the Apelin signaling pathway, vitamin D receptor, and spermine oxidase in skeletal muscle of middle-aged individuals, with these increases positively correlated with enhanced maximal oxidative phosphorylation capacity (McKenna et al., 1985). These findings suggest that resistance training may concurrently activate anabolic signaling and mitochondrial respiratory function, contributing to regulation of energy metabolic efficiency through modulation of age-related biomarkers. In addition, regulation of mitochondrial function by resistance training may occur indirectly *via* the endoplasmic reticulum unfolded protein response. One study found that 8 weeks of resistance training significantly activated PERK/ATF4 and IRE1/XBP1 UPR pathways in peripheral blood mononuclear cells of older adults, while upregulating PGC-1α and the mitochondrial fusion protein Mfn1, without altering levels of mitophagy markers PINK1/Parkin or Bnip3/Nix (Estébanez et al., 2019). These findings suggest that resistance training may participate, at least in part, in maintaining mitochondrial function through UPR-mediated signaling pathways.

Overall, the principal contribution of resistance training lies in optimizing myocellular structural integrity and improving local proteostasis and redox environment. In mild-to-moderate sarcopenia, its effects on the mitochondrial system are typically reflected in supporting and stabilizing existing mitochondrial function rather than robustly driving mitochondrial biogenesis (Springer-Sapp et al., 2025). Compared with endurance training or HIIT, resistance training has not consistently demonstrated significant increases in mitochondrial content or oxidative phosphorylation capacity across most studies. Thus, within the MQC network, its role is better characterized as providing structural and metabolic support for maintenance of mitochondrial homeostasis. In multimodal exercise interventions, the value of resistance training lies in consolidating the structural foundation of myocytes, preserving muscle mass and strength, and improving local metabolic and antioxidant status, thereby offering structural and functional synergy for mitochondrial remodeling induced by other training modalities such as endurance or interval training (Mesquita et al., 2020) (Table 2).

**TABLE 2:** Comparison of mechanisms by which different exercise modalities regulate aging-related mitochondrial quality control.

Exercise modality	MQC-related regulatory focus	Exercise-induced molecular changes	Functional outcomes	Study subjects	Level of evidence	References
Endurance Training	Short-term adaptation: fusion-dominant remodeling	↑ Fusion: MFN2, OPA1; ↑ Biogenesis: Complex III and V ↑↑, mitochondrial volume density (MitoVD) ↑; ↑ Mitophagy initiation: PINK1, BNIP3; Stable fission: DRP1 unchanged, Parkin unchanged	↑ MitoVD; improved VO_2_peak and oxidative capacity	Humans	Level II	Arribat et al. (2019)
	Long-term adaptation: mitophagy-dominant remodeling (different training durations within same study)	↑ Mitophagy: Parkin, BCL2L13, VDAC1; ↑ Fusion: MFN1, MFN2; ↓ Fission: DRP1 ↓, DRP1-Ser637 phosphorylation ↑	Reduced body fat; optimal VO_2_peak and oxidative capacity	Humans	Level II	Arribat et al. (2019)
	ADP sensitivity, ROS regulation, and network integrity	↓ Mitochondrial fragmentation; ↑ submaximal ADP-stimulated respiration; ↓ H_2_O_2_ emission; ↑ GSH	Improved ADP sensitivity; reduced ROS emission; enhanced antioxidant capacity; reversal of age-related mitochondrial fragmentation	Mice	Level III	Halling et al. (2019)
	PGC-1α signaling and mitochondrial biogenesis	↑ PGC-1α mRNA and nuclear protein; ↑ TFAM, cytochrome c, mtDNA content; ↑ p-AMPK, p-p38 MAPK, SIRT1, p-CREB and CREB DNA-binding activity	Reversal of aging-associated decline in mitochondrial content and function	Rats	Level III	Kang et al. (2013)
	Microenvironmental support (angiogenic adaptation)	↑ Capillary contacts in type I/IIA/IIB fibers; ↓ proportion of type IIB fibers; exercise-induced VEGF secretion (young > old); resting VEGF (old > young)	Preserved angiogenic capacity in elderly women; ↑ VO_2_max	Humans	Level II	Gavin et al. (2015)
	Mitochondrial respiration and oxidative stress regulation	↑ DNMT3A; ↓ ALDH1L1 mRNA/protein; ↓ NADPH, ↓ NOX activity, ↓ ROS (H_2_O_2_); ↑ SDH activity; ↑ mitochondrial respiration	Maintenance of skeletal muscle oxidative capacity and endurance performance	Mice	Level III	Damal Villivalam et al. (2021)
	NAMPT/NAD^+^ salvage pathway	↑ NAMPT protein	Reversal of age-related decline in skeletal muscle NAMPT; improved mitochondrial function	Humans	Level II	de Guia et al. (2019)
	SIRT3–miR-7 antioxidant metabolic axis	↑ SIRT3 protein; ↑ SOD2 protein; ↑ SIRT1 mRNA; ↑ FOXO1 mRNA; ↓ miR-7	Enhanced mitochondrial fatty acid oxidation and ATP synthesis efficiency; strengthened antioxidant defense; suppressed ROS accumulation	Humans	Level II	Koltai et al. (2018)
	Mitochondria–ER contact sites (MERCs) structure and composition	↑ MERC length; ↑ mitochondrial–SR MERC coverage; ↑ MERC number per mitochondrion; ↑ mitochondrial Ca^2+^ uptake rate and retention capacity; ↓ H_2_O_2_ emission	Restored relaxation kinetics; reduced fatigue susceptibility; improved mitochondrial Ca^2+^ handling; reduced ROS emission	Mice	Level III	Allen et al. (2025)
	Mitochondrial dynamics and MQC (fission–fusion–mitophagy axis); OxPhos and mitochondrial metabolism	↑ Fis1, Mfn2, OPA1 proteins; ↑ mitochondrial Parkin translocation; ↑ Beclin1 and Gabarap mRNA; ↑ mtDNA/nDNA ratio	Maintenance of MQC; enhanced segregation and clearance of damaged mitochondria; improved network plasticity; compensation for age-related mitochondrial decline	Humans	Level II	Balan et al. (2019)
	Parkin-mediated mitochondrial turnover (mitophagy–biogenesis coordination)	↑ Total Parkin; ↑ mitochondrial Parkin localization; ↑ PGC-1α; ↓ PARIS; ↑ COX I/II/IV; ↑ TFAM; ↑ COX activity; ↑ mitochondrial yield; ↓ p-ACC	Increased mitochondrial content and function; reduced acute exercise-induced mitophagy flux	Mice	Level III	Chen et al. (2018b)
	Mitochondrial function and innate immune signaling (NLRP3 inflammasome)	↑ Mitochondrial respiration; partial restoration of mitochondrial content (COX IV/COX I); ↓ NLRP3, caspase-1 p20, BAX, GSDMD, STING	Partial reversal of age-related low-grade inflammation; improved respiration; maintenance/increase of muscle mass (TA, GAST, SOL); improved acute endurance performance	Mice	Level III	Khemraj et al. (1985)
High-Intensity Interval Training (HIIT)	PGC-1α transcriptional regulation and p38 MAPK-mediated acute biogenic response	↑ p-p38 MAPK; ↑ PGC-1α mRNA; ↑ COX IV mRNA/protein; ↑ mtDNA content	Activation of mitochondrial biogenesis signaling; improved mitochondrial marker expression	Humans	Level II	Cobley et al. (2012)
	UPRmt and integrated stress response (ISR) signaling	↑ p-eIF2α; ↑ DDIT3, HSPD1, HSPE1, ATF3, FGF21, PPP1R15A mRNA; ↑ ULK1-Ser556 phosphorylation; mitochondrial ultrastructural disruption; mitophagosome formation	Induction of mitochondrial stress response and activation of UPRmt/ISR/MQC pathways	Humans	Level II	Botella et al. (2025)
	AMPK–biogenesis–mitophagy–supercomplex assembly axis	↑ AMPK pathway proteins; ↑ biogenesis and mitophagy markers; ↑ SOD2; ↑ circulating irisin; ↑ fusion proteins; ↑ mitochondrial supercomplex assembly; ↑ IL-15 and OPA1 mRNA	AMPK activation; enhanced biogenesis and mitophagy; promoted mitochondrial supercomplex formation; improved mitochondrial function in aged soleus	Rats	Level III	Han et al. (2022)
	Mitonuclear imbalance–UPRmt–biogenesis axis	↑ MTCO1/SDHA ratio; ↑ Yme1L1 and LONP1; ↑ NRF1, TFAM, VDAC; ↑ citrate synthase; ↑ mt-ND1, mt-CytB (mt-D-loop trend)	Improved mitochondrial proteostasis; increased biogenesis and mtDNA copy number in aged skeletal muscle	Mice	Level III	Co et al. (2021)
	SIRT3–biogenesis–basal autophagy activity	↑ SDH, COX IV, SIRT3, ALDH2; ↑ LC3-II, LC3-II/I ratio, ATG-3, Beclin-1	Improved endurance capacity, grip strength, lactate clearance; enhanced oxidative capacity; increased cardiac and slow-twitch muscle autophagy	Rats	Level III	Li et al. (2018)
	Lactate-mediated hippocampal MQC regulation	↑ Blood and hippocampal lactate; ↑ MCT1, MCT4; ↑ BDNF, ATP; ↑ COX5b mRNA; ↑ OPA1, MFN1/2; ↓ DRP1, FIS1; ↑ PGC-1α; ↑ mtDNA copy number	Enhanced hippocampal mitochondrial function; increased BDNF expression; improved brain function	Mice; Cells	Level III	Hu et al. (2021)
Resistance Training	Antioxidant enzyme system	↑ SOD1, SOD2, CAT; ↑ HSP60; ↑ CAT and TAC activity; ↓ GPX activity; ↓ 4-HNE	Enhanced antioxidant enzyme activity; reduced lipid peroxidation; improved redox status in elderly skeletal muscle	Humans	Level II	Mesquita et al. (2021)
	Mitochondrial respiration and H_2_O_2_ emission	↓ H_2_O_2_ emission rate	Improved mitochondrial respiratory capacity; reduced H_2_O_2_ emission; enhanced mitochondrial efficiency and redox balance	Humans	Level II	Flensted-Jensen et al. (2026)
	Indirect regulation *via* PGC-1α isoform specificity	↑ PGC-1α4; ↑ IGF-1; ↓ myostatin	Coordinated enhancement of mitochondrial function and muscle mass	Humans; Mice	Level I	Ruas et al. (2012)
	Integrated regulation of, ETC., complexes, fusion, biogenesis, and mitophagy	↑ Complex I–V proteins; ↑ Mfn1, Mfn2, Opa1, Drp1; no significant change in PGC-1α/TFAM/PINK1/Parkin; acute ↑ NRF1	Increased OxPhos protein content; promoted mitochondrial dynamic remodeling (inferred)	Humans	Level II	Mesquita et al. (2020)
	mtDNA methylome remodeling	159/254 CpG sites hypomethylated; enrichment in D-loop region; ↑ transcription of H- and L-strand genes; ↑ Complex III and IV proteins	Improved mitochondrial transcriptional function; increased respiratory chain protein expression	Humans	Level II	Ruple et al. (2021)
	Mitochondrial respiration and muscle-derived signaling	↑ CI + II OxPhos, ETS capacity, Vmax; ↑ plasma apelin; ↑ Apln, Aplnr, Vdr, Smox mRNA	Enhanced mitochondrial respiration; increased muscle strength; upregulation of targets linked to muscle mass and oxidative function	Humans	Level II	McKenna et al. (1985)
	Mitochondrial biogenesis, dynamics, and ER stress–UPR signaling	↑ PGC-1α, Mfn1; unchanged Drp1, PINK1/Parkin, Bnip3/Nix; ↑ p-PERK, p-IRE1, ATF4, XBP1	Activation of UPR (PERK/ATF4 and IRE1/XBP1 pathways); enhanced biogenesis and fusion; no activation of mitophagy	Humans	Level I	Estébanez et al. (2019)

## 6 Differences in mitochondrial quality control plasticity between healthy and pathological aging

In older adults without a substantial burden of chronic disease, skeletal muscle mitochondrial respiratory capacity, mitochondrial protein synthesis rates, and mitochondrial content are typically reduced compared with young adulthood. However, current evidence suggests that despite this functional decline, the overall structural coordination of the mitochondrial quality control (MQC) network remains largely preserved. In other words, the coupling among mitochondrial biogenesis, dynamic remodeling, and autophagic clearance is not completely disrupted. Some individuals are still able to maintain a relatively stable balance between mitochondrial renewal and removal under basal conditions. During midlife, partial compensatory mitochondrial biogenesis may help sustain relative metabolic stability; although this compensatory capacity gradually weakens in later life, it is not entirely lost. Regular exercise, as a key intervention validating MQC plasticity, has been shown to partially reactivate energy-sensing and biogenesis-related pathways, promoting coordination between mitochondrial renewal and autophagic processes, thereby improving mitochondrial functional status to a certain extent (Zhang et al., 2020),This residual plasticity may be associated with the preserved functionality of antioxidant defense systems.

Specifically, although aging is accompanied by reduced expression and activity of NRF2, exercise stimuli can still activate the NRF2 pathway within a certain range, promoting expression of downstream antioxidant enzymes such as catalase and superoxide dismutase. This reduces oxidative stress burden and helps maintain mitochondrial homeostasis to some degree (Angulo et al., 2020). In healthy aging, alterations in the MQC system more closely resemble a “reduced response amplitude” rather than a “loss of response mechanisms.” This is characterized by diminished sensitivity to acute stimuli and attenuated signal amplification, while core regulatory axes retain activation potential (Guo et al., 2023; Limanaqi et al., 2025). Studies have shown that aged skeletal muscle exhibits blunted signal transduction responses to acute exercise, including reduced activation of kinases such as AMPK, p38 MAPK, and CaMK, along with lower transcriptional induction of PGC-1α. Increased methylation of the PGC-1α promoter region and alterations in the transcriptional regulatory environment may contribute to this phenomenon. Importantly, despite attenuated signaling responses, long-term regular training can still partially improve mitochondrial functional indices. Even individuals initiating exercise later in life may demonstrate partial restoration of mitochondrial content or respiratory capacity, although the magnitude of adaptation is generally smaller than in younger individuals and may require longer training duration (Memme et al., 2021).

In contrast, pathological aging states such as frailty, sarcopenia, and metabolic dysfunction are more frequently accompanied by coordinated impairment across multiple MQC pathways, with a shift from decline in isolated components to network-level dysfunction. In some older adults with sarcopenia, increased mitochondrial DNA mutation burden, reduced respiratory capacity, and elevated reactive oxygen species production have been detected. Attenuated NRF2 function and hypermethylation of its promoter region have been reported in certain studies, further weakening baseline antioxidant defenses (Zhang et al., 2022). Concurrently, PINK1/Parkin-mediated mitophagy activity appears reduced in some populations. Although compensatory upregulation of BNIP3 or NIX expression may occur, such compensation does not necessarily translate into improved clearance efficiency, suggesting impairment of autophagic flux integrity.

In pathological aging, exercise interventions can still activate AMPK-, SIRT1-, and PGC-1α-related pathways and induce upregulation of certain autophagy markers. However, in individuals with sarcopenia, the magnitude, duration, and stability of mitochondrial functional improvements are often inferior to those observed in healthy older adults (Springer-Sapp et al., 2025; Huang et al., 2025; Marzetti et al., 2025b). This discrepancy indicates that once structural imbalance has emerged within the MQC network, exercise stimuli alone may be insufficient to fully restore coordination. Similar stratified differences are observed in other high–energy-demand tissues. For example, in the nervous system, diminished mitochondrial quality control function is associated with metabolic abnormalities and may contribute to neurodegenerative processes (Na et al., 2025); In vascular endothelium, analogous alterations have been reported: FUNDC1-mediated mitophagy declines with age in coronary endothelial cells, and under pathological conditions, even 4 weeks of regular swimming training shows markedly reduced protection against myocardial ischemia–reperfusion injury (Ye et al., 2020).

Overall, the fundamental distinction between healthy and pathological aging in terms of mitochondrial quality control lies in differences in network coordination capacity and reserve plasticity. While healthy aging is characterized by reduced functional levels but preserved responsiveness of core regulatory axes, pathological aging more often involves weakened antioxidant defenses, substantive decline in autophagic regulation, and increased heterogeneity in exercise responsiveness. Therefore, when evaluating exercise-induced MQC adaptations and their potential biomarker implications, distinguishing between healthy and pathological aging is methodologically essential. Without stratified analyses, mitochondrial phenotypes and exercise response patterns across distinct aging trajectories may be conflated, potentially obscuring mechanistic interpretation and accurate assessment of intervention efficacy.

## 7 Conclusion and perspectives

During aging, the mitochondrial quality control system undergoes multilayered regulatory alterations, including reduced mitochondrial biogenesis capacity, increased network fragmentation, diminished efficiency of selective autophagy, and weakened proteostatic regulation. Collectively, these changes disrupt energy metabolic homeostasis and are associated with chronic low-grade inflammation and multisystem functional decline. Based on current evidence, this review proposes that regular exercise can positively modulate these processes to a certain extent. Mechanisms include activation of AMPK/SIRT1/PGC-1α–related pathways, regulation of fusion and fission protein expression, modulation of PINK1/Parkin-mediated autophagy, and improvement of the protein folding and repair microenvironment. However, most mechanistic insights derive from basic or short-term intervention studies, and their generalizability across age groups and disease states in humans requires further validation.

Different exercise modalities exhibit distinct emphases in regulating mitochondrial homeostasis. Endurance training is more closely associated with metabolic adaptation and mitochondrial renewal; high-intensity interval training induces robust stress responses and remodeling signals; resistance training is more strongly linked to structural maintenance and functional optimization. In practical applications, rational combination of different exercise forms may help balance renewal capacity, structural integrity, and functional output. Nevertheless, long-term outcomes require confirmation through rigorous evidence-based evaluation.

Given that age-related mitochondrial dysregulation involves complex multi-pathway networks, single interventions are unlikely to address all components. Some studies suggest potential advantages of phased or combined training strategies in improving aging-related functional indices (Zhao and Gao, 2024; Sterczala et al., 2024). For example, endurance training may sustain baseline metabolic adaptation, periodic high-intensity stimuli may enhance stress responsiveness, and resistance training may preserve muscle mass and strength reserves. Theoretically, such integrated approaches may simultaneously influence biogenesis, autophagy, and anabolic signaling (Liu et al., 2021). However, safety thresholds vary according to age, fitness level, and comorbid conditions. Appropriate training intervals and adequate intake of protein and micronutrients are important for minimizing risk and improving adherence (Zhang et al., 2025). Moreover, age-related reductions in signaling sensitivity underscore the need for individualized, stratified exercise prescriptions rather than uniform protocols.

In recent years, various metabolic regulators termed “exercise mimetics” have emerged as potential research avenues for individuals with limited exercise capacity. Compounds such as urolithin A, NAD^+^ precursors, and certain AMPK modulators have demonstrated regulatory potential for mitophagy or energy metabolism in experimental or early clinical studies. However, there is currently insufficient evidence to support their ability to replicate the integrated multisystem effects of exercise across neural, cardiovascular, and muscular systems. At present, these agents should be regarded as potential adjunctive strategies rather than equivalent substitutes for exercise. Future research should further explore synergistic effects between exercise and nutritional or pharmacological interventions, incorporating stratification based on baseline mitochondrial function (Picca et al., 2023b; Singh et al., 2022).

From a translational perspective, several key challenges remain. First, many mechanistic findings derive from cellular or animal models whose metabolic characteristics differ inherently from humans. Second, substantial heterogeneity in exercise intensity, frequency, and duration complicates precise definition of dose–response relationships (Alldritt et al., 2025). Methodologically, there is no unified standard for assessing mitophagic flux, dynamics-related protein expression, or mitochondrial respiratory function, limiting cross-platform comparability. Individual factors—including age, sex, baseline metabolic state, and comorbidities—may significantly influence mitochondrial responses to exercise (Tang et al., 2024).

Clinically, clearer associations must be established among feasibility, safety, and efficacy of exercise interventions. Large-scale, long-term randomized controlled trials using ecologically valid exercise prescriptions and systematically quantifiable MQC-related biomarkers are warranted. Advanced molecular biology techniques may help bridge mechanistic research and clinical outcomes, deepening understanding of exercise-mediated MQC regulation. Ethical and practical considerations require cautious evaluation of high-intensity training in frail or very old populations, along with exploration of safe multimodal intervention strategies for individuals with multimorbidity, aiming to improve mitochondrial homeostasis without inducing excessive stress responses.

In addition, nutritional and supplementation strategies that modulate mitochondrial homeostasis merit systematic evaluation. Certain antioxidants, mitochondria-targeted molecules, or metabolic precursors have demonstrated potential to improve redox status or autophagy-related markers in experimental studies (Broome et al., 2024; Marcangeli et al., 2022). Future investigations should apply rigorous dose control, long-term follow-up, and safety monitoring to assess their true additive value when combined with exercise interventions. Technically, development of integrated assessment frameworks encompassing mitochondrial function, dynamics, and autophagic flux, along with reproducible and quantifiable circulating biomarker detection methods, will enhance comparability and clinical feasibility.

Overall, regular exercise remains one of the most evidence-supported and widely applicable interventions for partially mitigating age-related mitochondrial homeostatic imbalance. Nonetheless, current research is limited by model selection, measurement methodologies, and clinical endpoints. Long-term, double-blind, randomized human trials using lifespan as the ultimate endpoint pose substantial design and implementation challenges (Le Couteur et al., 2024). Future efforts should prioritize establishment of standardized evaluation systems, optimization of stratified precision exercise prescriptions, and integration with long-term functional outcomes. Only upon this foundation can the role of exercise in aging and chronic disease management be supported by more robust scientific evidence.
